# Efficacy and safety of anticoagulants for postoperative thrombophylaxis in total hip and knee arthroplasty: A PRISMA-compliant Bayesian network meta-analysis

**DOI:** 10.1371/journal.pone.0250096

**Published:** 2021-06-17

**Authors:** Tailai He, Fei Han, Jiahao Wang, Yihe Hu, Jianxi Zhu

**Affiliations:** 1 Department of Orthopaedic Surgery, Xiangya Hospital, Central South University, Changsha, China; 2 Department of Cardiothoracic Surgery, Xiangya Hospital, Central South University, Changsha, China; 3 Hunan Key Laboratary of Aging Biology, Xiangya Hospital, Central South University, Changsha, China; Kurume University School of Medicine, JAPAN

## Abstract

**Objective:**

To search, review, and analyze the efficacy and safety of various anticoagulants from randomized clinical trials (RCTs) of anticoagulants for THA and TKA.

**Design:**

PRISMA-compliant Bayesian Network Meta-analysis.

**Data sources and study selection:**

The databases of The Medline, Embase, ClinicalTrial, and Cochrane Library databases were searched until March 2017 for RCTs of patients undergoing a THA or TKA.

**Main outcomes and measures:**

The primary efficacy measurement was the venous thromboembolism Odds ratio (OR). The safety measurement was the odds ratio of major or clinically relevant bleeding. OR with 95% credibility intervals (95%CrIs) were calculated. Findings were interpreted as associations when the 95%CrIs excluded the null value.

**Results:**

Thirty-five RCTs (53787 patients; mean age range, mostly 55–70 years; mean weight range, mostly 55–90 kg; and a higher mean proportion of women than men, around 60%) included the following Anticoagulants categories: fondaparinux, edoxaban, rivaroxaban, apixaban, dabigatran, low-molecular-weight heparin, ximelagatran, aspirin, warfarin. Anticoagulants were ranked for effectiveness as follows: fondaparinux (88.89% ± 10.90%), edoxaban (85.87% ± 13.34%), rivaroxaban (86.08% ± 10.23%), apixaban (68.26% ± 10.82%), dabigatran (41.63% ± 12.26%), low-molecular-weight heparin (41.03% ± 9.60%), ximelagatran (37.81% ± 15.87%), aspirin (35.62% ± 20.60%), warfarin (9.89% ± 9.07%), and placebo (4.56% ± 6.37%). Ranking based on clinically relevant bleeding events was as follows: fondaparinux (14.53% ± 15.25%), ximelagatran (18.93% ± 17.49%), rivaroxaban (23.86% ± 15.14%), dabigatran (28.30% ± 14.18%), edoxaban (38.76% ± 24.25%), low-molecular-weight heparin (53.28% ± 8.40%), apixaban (71.81% ± 10.92%), placebo (76.26% ± 14.61%), aspirin (86.32% ± 25.74%), and warfarin (87.95% ± 11.27%). No statistically significant heterogeneity was observed between trials.

**Conclusions and relevance:**

According to our results, all anticoagulant drugs showed some effectiveness for VTE prophylaxis. Our ranking indicated that fondaparinux and rivaroxaban were safer and more effective than other anticoagulant drugs for patients undergoing THA or TKA.

## Introduction

Total joint arthroplasty is generally regarded as a highly successful surgical intervention. However, venous thromboembolism (VTE), including lower- and upper-extremity deep-vein thrombosis (DVT) and pulmonary embolism (PE), represents a major complication of this surgery. VTE has a combined annual incidence of 1–2 events per 1,000 population in the United States [1]. Warfarin (a vitamin K antagonist) is established as an effective agent for VTE prophylaxis [2, 3]. However, its potential to cause bleeding limits its use in major orthopaedic surgeries such as total hip arthroplasty (THA) and total knee arthroplasty (TKA). Alternatively, subcutaneous low-molecular-weight heparin (LMWH) has been widely used for VTE prophylaxis in recent decades, with a relatively safe outcome [4, 5]. Furthermore, newer targeted oral anticoagulants such as Factor Xa inhibitors (apixaban [6–9], rivaroxaban [10–17], and edoxaban [18–20]) and direct thrombin inhibitors (dabigatran [21–25] and ximelagatran [26–28]) can circumvent these limitations because of their faster onset and, to date, fewer known drug interactions requiring modification of therapy.

Among the available anticoagulants, dabigatran etexilate (Pradaxa; Boehringer Ingelheim), rivaroxaban (Xarelto; Bayer), apixaban (Eliquis), xilamegatran, fondaparinux [29–33] (Aristra, GSK), aspirin [34], warfarin [35, 36], and LMWH (Enoxaparin, Delteparin) are widely used for prophylaxis against VTE in patients undergoing THA or TKA. Although phase II and III trials have been performed to evaluate the efficacy of the newer drugs compared with LMWH, the pivotal studies on these indications were mainly based on comparisons with LMWH or placebo, with no head-to-head comparisons between the new oral anticoagulants reported to date.

Previous meta-analyses compared efficacy and safety between new oral anticoagulants and enoxaparin [37, 38]. However, The RCTs included in these studies were very limited and did not include ximelagatran and classic anticoagulants, such as aspirin and warfarin. With the advantages of the network meta-analysis, we can incorporate a much wider rangeof anticoagulants and clinical trials, thus making our research results more comprehensive.

We performed a meta-analysis of data from randomized clinical trials (RCTs) of widely used anticoagulants for prophylaxis against VTE in patients undergoing THA or TKA. Using both direct and indirect Bayesian comparisons of the data [39, 40], we performed a head-to-head comparison of anticoagulants to evaluate their relative effectiveness and tolerability, including the rate of VTE events, death, and major or clinically relevant non-major bleeding during the follow up period.

## Methods

This is a network meta-analysis of various anticoagulants from randomized clinical trials (RCTs) of anticoagulants for THA and TKA. This meta-analysis is reported in accordance with the Preferred Reporting Items for Meta-Analyses (PRISMA).

### Data sources

An online systematic search was performed for eligible trials using the electronic databases of MEDLINE(PubMed), Scopus, Embase, ClinicalTrial, and Cochrane Library databases. In addition, the following websites were searched to retrieve unpublished and ongoing studies: Current Controlled Trials, ClinicalTrials.gov, and the World Health Organization International Clinical Trials Registry. The search was performed from database inception until March 2017.

### Search strategy

The Medline, Embase, ClinicalTrial, and Cochrane Library databases were searched using a combination of a series of logic keywords and text words related to anticoagulants, THA or TKA, and RCT. Key terms used in the search included extension or extended treatment or therapy; total hip arthroplasty (or THA, total hip replacement) or total knee arthroplasty (or TKA, total knee replacement);venous thromboembolism (or VTE) or deep vein thrombosis (or DVT) or pulmonary embolism (or PE); anticoagulant or anticoagulant agent; apixaban (or Eliquis); rivaroxaban (or Xarelto); edoxaban; dabigatran (or Pradaxa); ximelagatran (or melagatran); fondaparinux (or Arixtra); low-molecular-weight heparin (or LMWH, exoxaparin, or delteparin); aspirin; and warfarin (or vitamin K antagonist). The complete search used for Pubmed was: Search: (venous thromboembolism [MeSH Terms] OR VTE [Text Word] OR deep vein thrombosis [MeSH Terms] OR DVT [Text word] OR pulmonary embolism [MeSH Terms] OR PE [Text word]) AND (anticoagulant [MeSH Terms] OR anticoagulant agent [Text word] OR apixaban [Text word] OR Eliquis [Text word] OR rivaroxaban [Text word] OR Xarelto [Text word] OR edoxaban [Text word] OR Pradaxa [Text word] OR t ximelagatran [Text word] OR melagatran [Text word] OR fondaparinux [Text word] OR Arixtra [Text word] OR low-molecular-weight heparin [MeSH Terms] OR LMWH [Text word] OR exoxaparin [Text word] OR delteparin [Text word] OR aspirin [Text word] OR warfarin [Text word] OR vitamin K antagonist) OR (total hip arthroplasty [MeSH Terms] OR THA [Text word] OR total knee arthroplasty [MeSH Terms] OR TKA [Text word]) Filters: Clinical Trial.

### Selection criteria

**Fig 1:**
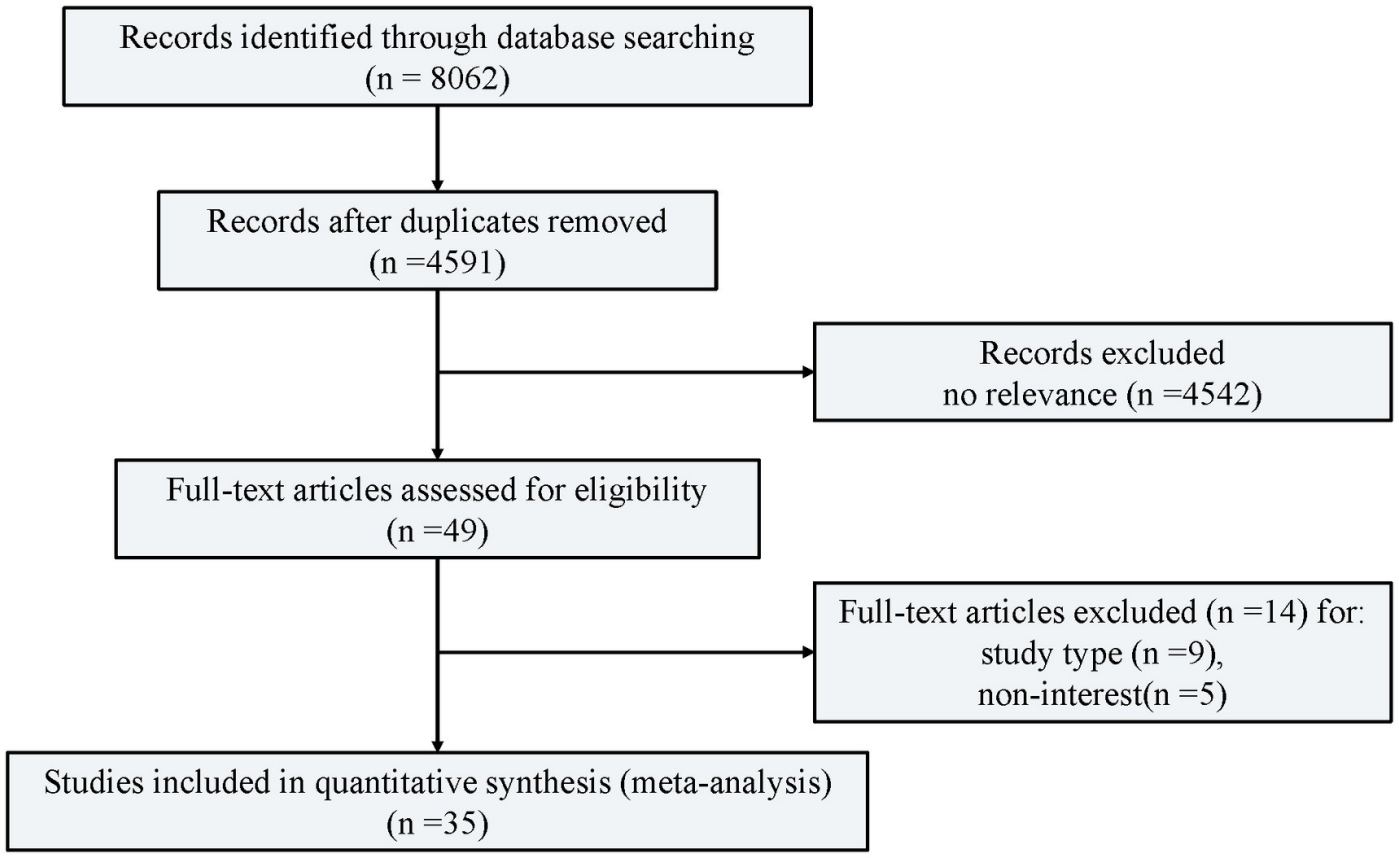
Flow diagram of study identification, screening, eligibility assessment, and inclusion. 35 randomised trials correspond to 71 groups because three-group studies were included in this multiple-treatments meta-analysis.

Studies selected (Fig 1) were RCTs that fulfilled the following inclusion criteria: (1) studies in adult patients undergoing a THA or TKA, regardless of the aetiology and type or size of prosthesis used; (2) studies with more than 100 patients; and (3) studies where the full text of the article was available. Exclusion criteria were (1) reviews, retrospective or observational studies, case reports, animal research, and studies without a case-control design; (2) studies on other types of CP and in patients previously diagnosed with other diseases that can cause VTE or bleeding; and (3) studies in patients with a mean age of less than 12 years or more than 80 years.

### Study selection and data extraction

Each citation was independently reviewed by two reviewers (J.W. and F.H.) according to a PRISMA flowchart (Fig 1). Citations were mostly excluded because of irrelevance, as determined by the title or abstract. For all other citations, both reviewers obtained the complete manuscript and evaluated it. Retrospective or non-randomized studies were excluded at this stage. Disagreement between the reviewers was resolved by consensus with a third reviewer (T.H.). Parameters including the author’s name, publication year, journal name, type of study, sample size, gender ratio, mean age and weight, type of surgery, dose and duration of anticoagulant drugs, other postoperative thromboprophylaxis, postoperative complications (VTE and bleeding), and duration of follow-up were evaluated.

### Quality and risk of bias assessment

The methodological quality of each component study was assessed using Jadad scoring [41]. We included only articles with Jadad scores ≥3. Reliability between reviewers was evaluated using the intra-class correlation coefficient (ICC).

### Data synthesis and analysis

A random effects Bayesian network meta-analysis was performed to compare the relative treatment effect of anticoagulants. A major advantage of network meta-analysis is that it allows the indirect comparison of interventions between primary trials. The meta-analysis was performed using WinBUGS software (version 1.4.3, MRC Biostatistics Unit, Cambridge, UK) and R version 3.0.2 (The R Foundation for Statistical Computing). Network meta-analysis is considered the most comprehensive approach to the comparison of multiple treatments [39], as it performs direct comparisons between two trials (A vs B) and indirect comparisons between trials with a common treatment (A vs C, using trials comparing A vs B and B vs C) [42]. The Markov chain Monte Carlo method was used to obtain the pooled effect sizes. Markov chains run simultaneously with different initial values chosen arbitrarily. Fifty thousand simulations were generated for each of the three sets of initial values. The first 10,000 simulations were regarded as the burn-in period and not used in the analysis. Pooled effect sizes were reported from the median of the posterior distribution, and the corresponding 95% credible intervals were applied using the 2.5th and 97.5th percentiles of the posterior distribution, which was similar to the conventional 95% CrIs.

We assessed the possibility of publication bias by constructing a funnel plot of each trial’s effect size against the standard error. Furthermore, we assessed funnel plot asymmetry using Begg tests, and defined significant publication bias as p value <0·05. To estimate the network inconsistency between the indirect and direct estimates in each closed loop, the absolute difference between the indirect and direct treatment effect estimates was calculated. Loops where the lower CI limit did not reach zero were considered a statistically significant inconsistency [43]. The fit of the model to the data was measured by calculating the posterior mean residual deviance. A model was considered to fit the data adequately when the mean of the residual deviance was similar to the number of data points. Sensitivity analysis was conducted to examine the impact of low methodological quality and small sample size on the overall effect size.

At the end of the study, we assessed efficacies and safeties between the anticoagulants and expressed these using placebo as reference. In each Markov chain Monte Carlo cycle, each agent was ranked from first to last according to the estimated effect size. These probabilities sum to one were displayed as histograms for each treatment and each rank. The anticoagulants were ranked for efficacy and safety according to their posterior probabilities. Probability values were summarized and reported as the surface under the cumulative ranking (SUCRA) [44]. The value of SUCRA ranged from 0 (worst treatment) to 1 (best treatment).

### Patient and public involvement

No patients or members of the public were involved in the present study. No patients were asked to advise on the interpretation or writing up of results. The results of the present research will be communicated to the relevant patient community.

## Results

A total of 35 RCTs were selected for network meta-analysis. The initial electronic database search identified 8,062 records, of which 8,027 were excluded after screening. First, 3471 citations were removed because of duplication. Next, 4542 publications were excluded based on the title or abstract because of irrelevancy. By subsequently scrutinizing the entire paper, 49 full-text papers remained. After excluding some heterogeneous studies, a total of 35 citations remained for analysis. Most trials were two-grouped studies and only one was three-grouped. Of these trials, one active comparator was usually LMWH. Patients had mean age ranged mostly 55–70 years, mean weight ranged mostly 55–90 kg, and higher mean proportion of women than men (around 60%). The basic characters of the trials are shown in Table 1. The quality of all trials were rated as good, which was assessed using Jadad scoring (≥3).

**Table 1:** Characteristics of trials included in the analysis.

Citation	Type of Intervention and Dose	Sample Size	Age Year	Weight Kg	Gender(M/F)	Surgery Type	Citation numbers
ADVANCE-1	Apixaban 2.5mg, bid, 10–14 days	1599	65.9	86.7	1212/1983	TKA	8
	Enoxaprin 30mg bid, 10–14 days	1596	65.7	86.7			
ADVANCE-2	Apixaban 2.5mg, bid, 10–14 days	1528	67	78	841/2216	TKA	7
	Enoxaprin 40mg od, 10–14 days	1529	67	78			
ADVANCE-3	Apixaban 2.5mg, bid, 28–35 days	2708	60.9	79.9	2526/2881	THA	6
	Enoxaprin 40mg od, 28–35 days	2699	60.6	79.5			
APROPOS	Apixaban 2.5mg, bid, 10–14 days	153	67.6	82.3	109/198	TKA	9
	Enoxaprin 30mg bid, 10–14 days	152	66.5	83.1			
RE-MODEL	Dabigatran 150, 220mg od, 6–10 days	1382	67.5	82.5	706/1370	TKA	23
	Enoxaparin 40mg od, 6–10 days	694	68	82			
RE-NOVATE	Dabigatran 150, 220mg od, 28–35 days	2309	64	79	1509/1954	THA	24
	Enoxaparin 40mg od, 28–35 days	1154	64	78			
RE-MOBILIZE	Dabigatran 150, 220mg od, 12–15 days	1728	66.1	88	1099/1497	TKA	25
	Enoxaparin 30mg bd, 12–15 days	868	66.3	88			
RE-NOVATEII	Dabigatran 220mg od, 28–35 days	1010	61.9	NR	1042/971	THA	22
	Enoxaparin 40mg od, 28–35 days	1003	62				
NCT00246025	Dabigatran 150, 220mg od, 6–10 days	255	71.8	NR	319/60	TKA	
	Placebo	124	71.3				
BISTRO-II	Dabigatran 150, 300mg od until venography	775	66.2	79	428/739	THA & TKA	21
	Enoxaparin 40mg od,until venography	392	65	79			
RECORD 1	Rivaroxaban 10mg od, 31–39 days	2209	63.1	78.1	1971/2462	THA	12
	Enoxaparin 40mg od, 31–39 days	2224	63.3	78.3			
RECORD 2	Rivaroxaban 10mg od, 31–39 days	1228	61.4	74.3	1139/1318	THA	13
	Enoxaparin 40mg od, 10–14 days	1229	61.6	75.2			
RECORD 3	Rivaroxaban 10mg od, 10–14 days	1220	67.6	80.1	781/1678	TKA	14
	Enoxaparin 40mg od, 10–14 days	1239	67.6	81.2			
RECORD 4	Rivaroxaban 10mg od, 11–15 days	1526	64.4	84.7	1060/1974	TKA	15
	Enoxaparin 30mg bid, 11–15 days	1508	64.7	84.4			
PROOF CONCEPT	Rivaroxaban 5, 10mg bid, 5–9 days	148	66.2	77.3	127/183	THA	
	Enoxaparin 40mg od, 5–9 days	162	64	79			
ODIXA KNEE	Rivaroxaban 10mg od, 5–9 days	103	67	86.4	84/123	TKA	17
	Enoxaparin 30mg bid, 5–9 days	104	66	89.3			
ODIXA HIP od	Rivaroxaban 10mg od, 5–9 days	142	64	75.6	109/190	THA	11
	Enoxaparin 40mg od, 5–9 days	157	65.6	74.9			
ODIXA HIP td	Rivaroxaban 5,10mg bid, 5–9 days	269	64.5	78	170/231	THA	10
	Enoxaparin 40mg od, 5–9 days	132	65	77			
Zou Y 2014	Rivaroxaban mg od, 14 days	102	63.5	NR	264/60	TKA	16
	Aspirin 100mg od, 14 days	112	65.7				
	Enoxaparin 40mg od, 14 days	110	62.7				
PENTAMAKS	Fondaparinux 2.5mg od, 5–9 days	517	67.5	89	427/607	TKA	29
	Enoxaprin 30mg bid, 5–9 days	517	67.5	88.4			
EPHESUS	Fondaparinux 2.5mg od, 5–9 days	1140	66	75	966/1307	THA	32
	Enoxaprin 40mg od, 5–9 days	1133	67	75			
PENTATHALON	Fondaparinux 2.5mg od, 5–9 days	1128	67	81	1078/1179	THA	33
	Enoxaprin 30mg bid, 5–9 days	1129	67	80			
Fuji T 2008	Fondaparinux 2.5mg od, 11–15 days	165	66.3	56.7	55/277	THA & TKA	31
	Placebo	167	66.4	57.6			
ALEXANDER G 2001	Fondaparinux 3.0mg od, 5–10 days	177	66	80	203/234	THA	30
	Enoxaparin 30mg od, 5–10 days	260	66	81			
Fuji T 2014	Edoxaban 30mg od,11–14 days	72	60.6	57.6	18/128	THA	19
	Enoxaparin 20mg bid, 11–14 days	74	58.9	56.7			
STARS E-3	Edoxaban 30mg od,11–14 days	299	72.6	59.6	120/474	TKA	20
	Enoxaparin 20mg bid, 11–14 days	295	72.1	60.7			
Fuji T 2010	Edoxaban 30mg od,11–14 days	103	71.4	60.7	47/158	TKA	18
	Placebo	102	70.6	61.2			
NCT01181167	Edoxaban 30mg od,11–14 days	255	62.8	NR	71/432	THA	
	Enoxaparin 20mg bid, 11–14 days	248	62.8				
EXTEND	Ximelagatran 24mg bid, 32–38 days	479	64.7	NR	440/518	THA	26
	Enoxaparin 40mg od, 32–38 days	479	63.9				
Colwell CW 2003	Ximelagatran 24mg bid, 7–12 days	782	64.5	80.5	749/808	THA	27
	Enoxaparin 30mg bid, 7–12 days	775	64	81			
EXPRESS	Ximelagatran 24mg bid, 8–11 days	1377	67	78	1051/1713	THA & TKA	28
	Enoxaparin 40mg od, 8–11 days	1387	67	79.1			
Fuji T(E)	Enoxaparin 40mg od, 14 days	154	61.8	55.9	43/276	THA & TKA	
	Placebo	165	65.4	56.6			
Hull RD 2000(1)	Delteparin 5000IU, od, 8 days	983	63.5	80.5	709/763	THA	35
	Warfarin 5-10mg, od, 8 days	489	63	80			
Hull RD 2000(2)	Delteparin 5000IU, od, 8 days	389	62.5	81	287/282	THA	36
	Warfarin 5-10mg, od, 8 days	180	63	81			
Anderson DR 2013	Delteparin 5000IU, od, 8–10 days	400	57.9	NR	444/341	THA	34
	Aspirin 81mg, od, 8–10 days	385	57.6				

**Fig 2:**
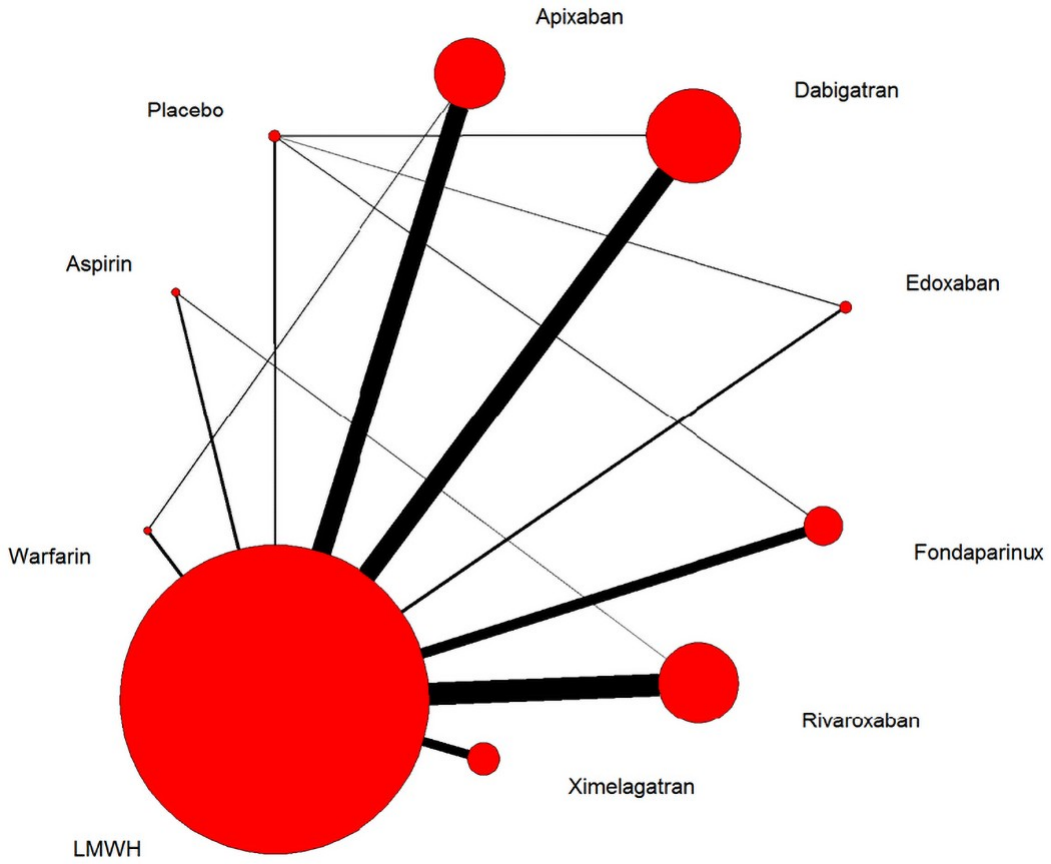
Network of eligible comparisons for the multiple-treatments meta-analysis. The width of the lines is proportional to the number of trials comparing every pair of treatments, and the size of every node is proportional to the number of randomised participants (sample size).

We established a network that included slightly different sets of studies (Fig 2), for which sensitivity analysis showed no significant heterogeneity. Of the 45 possible pair-wise comparisons between the 14 treatments, 14 have been studied directly in one or more trials for efficacy and safety.

**Fig 3:**
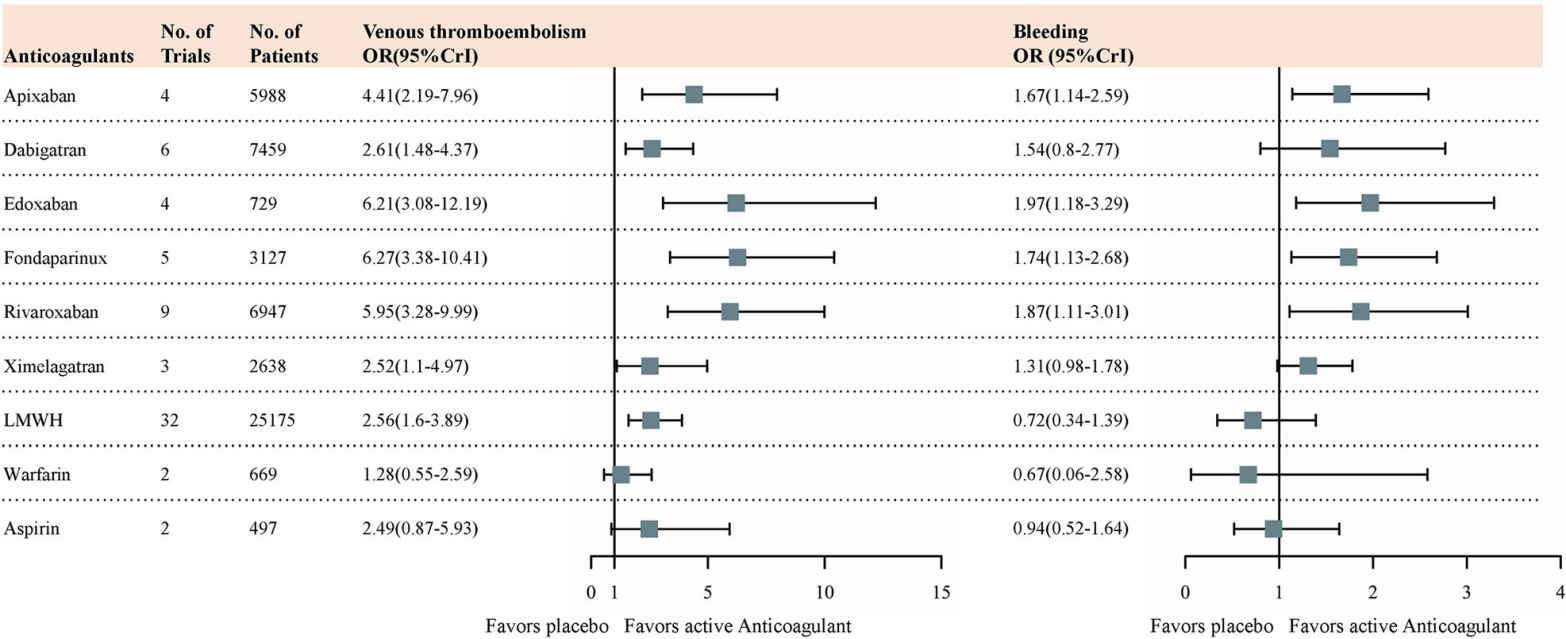
Forest plots of MTM results for efficacy outcomes and safety outcomes with placebo as reference compound. ORs higher than 1 favour active compound. MTM = multiple-treatments meta-analysis. OR = odds ratio. CrI = credibilty interval.

The Bayesian network meta-analysis results for the primary outcomes of interest were used for comparing RCTs. Based on the results of the Bayesian network, all anticoagulant agents showed some degree of efficacy compared with placebo (Fig 3). S1 Fig summarizes the results of the multiple-treatments meta-analyses for bleeding rate and thromboembolic events according to the network we established. For the efficacy evaluation, we selected the rate of DVT, which was the most used parameter for anticoagulant drugs. Among the available anticoagulants, Xa inhibitors such as fondaparinux (OR, 6.27 [95%CrI, 3.38 to 10.41]), edoxaban (OR, 6.21 [95%CrI, 3.08 to 12.19]), rivaroxaban (OR, 5.95 [95%CrI, 3.28 to 9.99]) and apixaban (OR, 4.41 [95%CrI, 2.19 to 7.96]) showed a relatively large effective size compared with the other anticoagulants. There is no obvious difference in effective size between direct thrombin inhibitors (dabigatran (OR, 2.61 [95%CrI, 1.48 to 4.37]) and ximelagatran (OR, 2.52 [95%CrI, 1.1 to 4.97])), LMWH (OR, 2.56 [95%CrI, 1.6 to 3.89]) and aspirin (OR, 2.49 [95%CrI, 0.87 to 5.93]). As a classic vitamin K antagonist, warfarin (OR, 1.28 [95%CrI, 0.55 to 2.59]) showed the minimum effective size among all available anticoagulants.

At the same time, we selected the rate of clinically relevant bleeding events as the representative parameter for side effects. In terms of the magnitude of the effect, warfarin (OR, 0.67 [95%CrI, 0.06 to 2.58]), LMWH (OR, 0.72 [95%CrI, 0.34 to 1.39]) and aspirin (OR, 0.94 [95%CrI, 0.52 to 1.64]) had significantly larger values than the other treatments as expected. However, even when it comes to safety assessment, Xa inhibitors (fondaparinux (OR, 1.74 [95%CrI,1.13 to 2.68]), edoxaban (OR, 1.97 [95%CrI, 1.18 to 3.29]), rivaroxaban (OR, 1.87 [95%CrI, 1.11 to 3.01]) and apixaban (OR, 1.67 [95%CrI, 1.14 to 2.59])) and direct thrombin inhibitors such as dabigatran (OR, 1.54 [95%CrI, 0.8 to 2.77]) and ximelagatran (OR, 1.31 [95%CrI, 0.98 to 1.78]) still showed a higher priority of safety.

No significant difference was observed between the direct and indirect comparisons, as shown in a Chaimani diagram (S2 Fig), indicating the coherence of data selected from different studies. The funnel plot results suggested that publication bias was not significant across the selected citations (S3 Fig). We utilized both fixed effects and random effects models and compared the differences between these two models. There was no obvious difference in parameters, indicating that the heterogeneity of citations was tolerable (supplementary random effects model: totresdev: 75.7904, pD: 50.0, DIC: 441.1; fixed effect model: totresdev: 84.1592, pD: 42.8, DIC: 442.3).

The SUCRA results (S4 Fig) show the ranking probability of all treatment regiments from the best treatment effect to the last. Treatments with a greater area in the histogram were associated with larger probabilities of better outcomes. According to our Bayesian network of therapeutic effect, the most efficacious treatments were fondaparinux (88.89% ±± 10.90%), edoxaban (85.87% ± 13.34%), rivaroxaban (86.08% ± 10.23%), apixaban (68.26% ± 10.82%), dabigatran (41.63% ± 12.26%), LMWH (41.03% ± 9.60%), ximelagatran (37.81% ± 15.87%), aspirin (35.62% ± 20.60%), warfarin (9.89% ± 9.07%), and placebo (4.56% ± 6.37%). The SUCRA histogram of different treatments is shown in S5 Fig.

Conversely, a lower SUCRA position for side effects indicated a higher priority of safety. Using the Bayesian network we constructed for clinically relevant bleeding rate, the detailed rank probabilities of each treatment were determined (S4 Fig). The histogram of treatments is shown in S6 Fig. The SUCRA ranking for clinically relevant bleeding events was as follows: fondaparinux (14.53% ± 15.25%), ximelagatran (18.93% ± 17.49%), rivaroxaban (23.86% ± 15.14%), dabigatran (28.30% ± 14.18%), edoxaban (38.76% ± 24.25%), LMWH (53.28% ± 8.40%), apixaban (71.81% ± 10.92%), placebo (76.26% ± 14.61%), aspirin (86.32% ± 25.74%), and warfarin (87.95% ± 11.27%).

**Fig 4:**
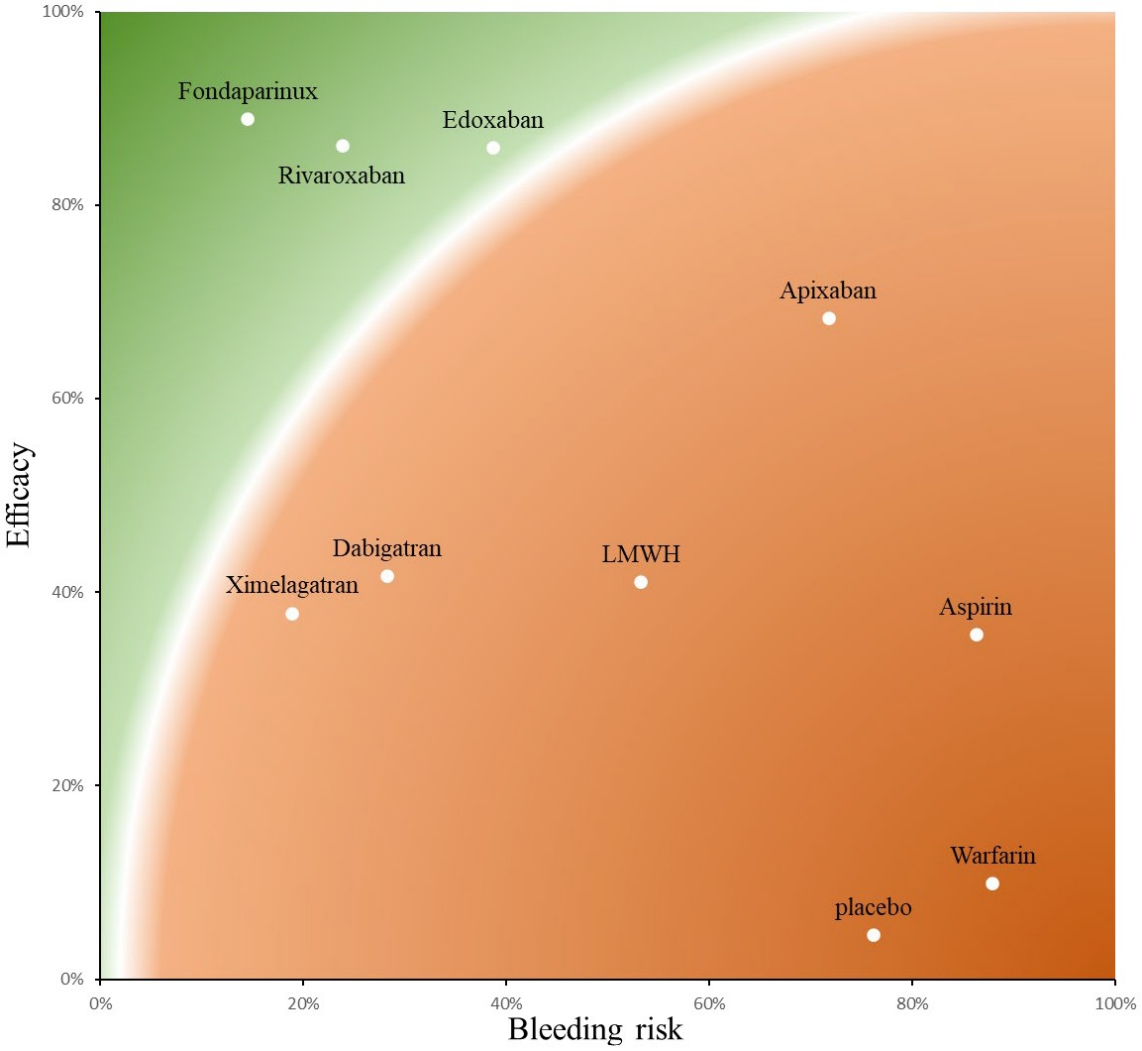
The anticoagulants were ranked for efficacy and safety according to their SUCRA score. Red color represents worst treatment and green represents best treatment in a qualitative approach. Treatments with a higher SUCRA position for VTE prophylaxis were associated with larger probabilities of better outcomes. Conversely, a lower SUCRA position for side effects (major or clinically relevant bleeding) indicated a higher priority of safety.

In summary, fondaparinux, rivaroxaban and edoxaban were among the most effective treatments, and fondaparinux, ximelagatran, and rivaroxaban were better than other anticoagulants in terms of safety. We ranked anticoagulants according to these two dimensions (Fig 4).

## Discussion

Our Bayesian network meta-analysis reviewed 9 anticoagulant agents for efficacy and safety in patients undergoing THA and TKA. To the best of our knowledge, this analysis comparing multiple anticoagulant drugs for these types of surgery include the most types of anticoagulants and the largest number of RCTs. We compiled evidence from direct and indirect comparisons to evaluate relative efficacy and safety parameters. Fondaparinux, edoxaban, and rivaroxaban were found to be the most effective anticoagulants for patients undergoing THA or TKA compared with the other drugs. In terms of safety, fondaparinux, ximelagatran, and rivaroxaban were the highest-ranked drugs for low prevalence of clinically relevant bleeding events. New oral anticoagulant drugs such as factor Xa inhibitors and direct thrombin inhibitors have a considerable improvement over the traditional oral or subcutaneous anticoagulants in terms of effectiveness and safety. Our findings indicate that, regardless of efficacy or safety at the last follow-up time point, fondaparinux and rivaroxaban were the most likely preferred drugs, and demonstrated the usefulness of network meta-analysis to compare the relative effectiveness and safety of different anticoagulant interventions. These results may benefit doctors, healthcare policymakers, and pharmaceutical companies involved in anticoagulation therapy. We excluded trials that were not properly blinded, had a small sample size, or were not sufficiently randomized. Moreover, we controlled for trial characteristics that could result in heterogeneity. Furthermore, Begg’s test indicated that publication bias was not significant across the included citations. There was no evidence of inconsistency between the direct and indirect comparisons according to the Chaimani and Higgins inconsistency tests.

New oral anticoagulant drugs confer multiple advantages compared with traditional oral or subcutaneous anticoagulants after major orthopaedic surgery such as THA or TKA. Fondaparinux and rivaroxaban are examples of newly developed direct factor Xa inhibitor and direct thrombin inhibitor, respectively. First, they exhibit higher anticoagulation activity than classical oral anticoagulants such as warfarin and aspirin [45, 46]. Second, they have been shown to be safer than warfarin, with fewer bleeding events, and do not require regular assessment of coagulation using tests such as the international normalized ratio (INR) [45]. Third, the use of oral anticoagulants after THA or TKA appears to be convenient and safe, with increased patient compliance, compared with LMWH. Given that that 35 days of anticoagulation is typically required following THA, subcutaneous injection of LMWH might not be feasible. Factor Xa inhibitors, with once-daily oral administration and no coagulation assessment, may be more acceptable to outpatients. Finally, unlike the traditional anticoagulant, warfarin, fondaparinux and rivaroxaban can be administrated at a convenient fixed dose. Nevertheless, an obvious limitation of Xa factor inhibitor is that there is no specific antidote available to reverse the effects of overdose. However, the risk of major bleeding events associated with these drugs is relatively low. Some reports have shown that recombinant activated Factor VIIa (rFVIIa) or Factor VIII inhibitor bypass activity (FEIBA) may counteract rivaroxaban overdose [45], although clinical data supporting this strategy are lacking.

The limitations of this study should also be addressed. Firstly, We identified a large pool of citations for the meta-analysis, from which considerable variation may derive. Variations in dosage, patient characteristics, surgery, and time point to final follow-up, for example, could contribute to heterogeneity. However, inconsistency was shown to be tolerable in the network meta-analysis. Secondly, although 35 long-term RCTs were retrieved, including approximately 53 787patients and studying many anticoagulant drugs, 2 classic anticoagulants (warfarin and aspirin) were still studied just in 2 trials and there were relatively few direct comparisons between anticoagulants and placebo. Thirdly, several included studies measured pain or functional parameters in a short term treatment courses. It is uncertain whether these effects may diminish over time. Fourth, this study focused only on the major parameters of VTE and clinically relevant bleeding events, without regarding secondary parameters. The measurement of other indices in a Bayesian network meta -analysis is challenging, and difficult to interpret. The SUCRA curve was used to estimate a ranking probability of comparative effectiveness and safety between the different anticoagulants, but it has limitations and the results should be interpreted with caution. Finally, this study shared some of the general limitations of all meta-analyses, in that it cannot discriminate between non-comparability of measures and outcomes across different studies. The inherent variations between different studies in terms of measurement and quantification could therefore not be addressed or completely eliminated [47].

## Conclusion

Our Bayesian network comparisons showed that all anticoagulant drugs had a certain level of effectiveness for VTE prophylaxis. Although further studies are needed to establish the optimal approach to the application of this treatment in practice, Our rankings clearly lend support to the use of fondaparinux or rivaroxaban were safer and more effective than other anticoagulant drugs for patients undergoing THA or TKA.

## Supporting information

S1 Fig The results of the multiple-treatments meta-analyses for bleeding rate and thromboembolic events. (TIF)

S2 Fig Chaimani diagram. No significant difference was observed between the direct and indirect comparisons.
(TIF)

S3 Fig Funnel plot. Publication bias was not significant across the selected citations.
(TIF)

S4 Fig The ranking probability of all treatment regiments from the best treatment effect to the last. (TIF)

S5 Fig The SUCRA histogram of each treatment for thromboembolic events. Treatments with a higher SUCRA position for VTE prophylaxis were associated with larger probabilities of better outcomes.
(TIF)

S6 Fig The SUCRA histogram of each treatment for clinically relevant bleeding rate. A lower SUCRA position for side effects (major or clinically relevant bleeding) indicated a higher priority of safety.
(TIF)

S1 File PRISMA 2009 flow diagram. (DOC)

S2 File PRISMA NMA checklist of items to include when reporting a systematic review involving a network meta-analysis. (DOCX)

S3 File (XLSX)

## References

[1] Silverstein MD, Heit JA, Mohr DN, et al. Trends in the incidence of deep vein thrombosis and pulmonary embolism: a 25-year population-based study. *Archives of internal medicine* 1998;158(6):585–93. doi: 10.1001/archinte.158.6.585 9521222

[2] Ost D, Tepper J, Mihara H, et al. Duration of anticoagulation following venous thrombolembolism—A meta-analysis. *Jama-Journal of the American Medical Association* 2005;294(6):706–15. doi: 10.1001/jama.294.6.706 16091573

[3] Geerts WH, Bergqvist D, Pineo GF, et al. Prevention of venous thromboembolism. *Chest* 2008;133(6):381S–453S. doi: 10.1378/chest.08-0656 18574271

[4] Hull RD, Pineo GF, Francis C, et al. Low-molecular-weight heparin prophylaxis using dalteparin extended out-of-hospital vs in-hospital warfarin/out-of-hospital placebo in hip arthroplasty patients—A double-blind, randomized comparison. *Archives of Internal Medicine* 2000;160(14):2208–15. doi: 10.1001/archinte.160.14.2208 10904465

[5] Hull RD, Pineo GF, Francis C, et al. Low-molecular-weight heparin prophylaxis using dalteparin in close proximity to surgery vs warfarin in hip arthroplasty patients—A double-blind, randomized comparison. *Archives of Internal Medicine* 2000;160(14):2199–207. doi: 10.1001/archinte.160.14.2199 10904464

[6] Lassen MR, Gallus A, Raskob GE, et al. Apixaban versus enoxaparin for thromboprophylaxis after hip replacement. *N Engl J Med* 2010;363(26):2487–98. doi: 10.1056/NEJMoa1006885 [published Online First: 2010/12/24] 21175312

[7] Lassen MR, Raskob GE, Gallus A, et al. Apixaban versus enoxaparin for thromboprophylaxis after knee replacement (ADVANCE-2): a randomised double-blind trial. *Lancet* 2010;375(9717):807–15. doi: 10.1016/S0140-6736(09)62125-5 [published Online First: 2010/03/09] 20206776

[8] Lassen MR, Raskob GE, Gallus A, et al. Apixaban or enoxaparin for thromboprophylaxis after knee replacement. *N Engl J Med* 2009;361(6):594–604. doi: 10.1056/NEJMoa0810773 [published Online First: 2009/08/07] 19657123

[9] Eriksson BI, Turpie AG, Lassen MR, et al. A dose escalation study of YM150, an oral direct factor Xa inhibitor, in the prevention of venous thromboembolism in elective primary hip replacement surgery. *J Thromb Haemost* 2007;5(8):1660–5. doi: 10.1111/j.1538-7836.2007.02644.x [published Online First: 2007/08/01] 17663737

[10] Eriksson BI, Borris LC, Dahl OE, et al. Dose-escalation study of rivaroxaban (BAY 59–7939)—an oral, direct Factor Xa inhibitor—for the prevention of venous thromboembolism in patients undergoing total hip replacement. *Thromb Res* 2007;120(5):685–93. doi: 10.1016/j.thromres.2006.12.025 [published Online First: 2007/02/13] 17292948

[11] Eriksson BI, Borris LC, Dahl OE, et al. A once-daily, oral, direct Factor Xa inhibitor, rivaroxaban (BAY 59–7939), for thromboprophylaxis after total hip replacement. *Circulation* 2006;114(22):2374–81. doi: 10.1161/CIRCULATIONAHA.106.642074 [published Online First: 2006/11/23] 17116766

[12] Eriksson BI, Borris LC, Friedman RJ, et al. Rivaroxaban versus enoxaparin for thromboprophylaxis after hip arthroplasty. *N Engl J Med* 2008;358(26):2765–75. doi: 10.1056/NEJMoa0800374 [published Online First: 2008/06/27] 18579811

[13] Kakkar AK, Brenner B, Dahl OE, et al. Extended duration rivaroxaban versus short-term enoxaparin for the prevention of venous thromboembolism after total hip arthroplasty: a double-blind, randomised controlled trial. *Lancet* 2008;372(9632):31–9. doi: 10.1016/S0140-6736(08)60880-6 [published Online First: 2008/06/28] 18582928

[14] Lassen MR, Ageno W, Borris LC, et al. Rivaroxaban versus enoxaparin for thromboprophylaxis after total knee arthroplasty. *N Engl J Med* 2008;358(26):2776–86. doi: 10.1056/NEJMoa076016 [published Online First: 2008/06/27] 18579812

[15] Turpie AG, Lassen MR, Davidson BL, et al. Rivaroxaban versus enoxaparin for thromboprophylaxis after total knee arthroplasty (RECORD4): a randomised trial. *Lancet* 2009;373(9676):1673–80. doi: 10.1016/S0140-6736(09)60734-0 [published Online First: 2009/05/05] 19411100

[16] Zou Y, Tian S, Wang Y, et al. Administering aspirin, rivaroxaban and low-molecular-weight heparin to prevent deep venous thrombosis after total knee arthroplasty. *Blood Coagul Fibrinolysis* 2014;25(7):660–4. doi: 10.1097/MBC.0000000000000121 [published Online First: 2014/04/04] 24695091

[17] Turpie AG, Fisher WD, Bauer KA, et al. BAY 59–7939: an oral, direct factor Xa inhibitor for the prevention of venous thromboembolism in patients after total knee replacement. A phase II dose-ranging study. *J Thromb Haemost* 2005;3(11):2479–86. doi: 10.1111/j.1538-7836.2005.01602.x [published Online First: 2005/10/26] 16241946

[18] Fuji T, Fujita S, Kawai Y, et al. Efficacy and safety of edoxaban versus enoxaparin for the prevention of venous thromboembolism following total hip arthroplasty: STARS J-V. *Thromb J* 2015;13:27. doi: 10.1186/s12959-015-0057-x [published Online First: 2015/08/14] 26269694 PMC4534125

[19] Fuji T, Wang CJ, Fujita S, et al. Safety and efficacy of edoxaban, an oral factor xa inhibitor, for thromboprophylaxis after total hip arthroplasty in Japan and Taiwan. *J Arthroplasty* 2014;29(12):2439–46. doi: 10.1016/j.arth.2014.05.029 [published Online First: 2014/07/23] 25047458

[20] Fuji T, Wang CJ, Fujita S, et al. Safety and efficacy of edoxaban, an oral factor Xa inhibitor, versus enoxaparin for thromboprophylaxis after total knee arthroplasty: the STARS E-3 trial. *Thromb Res* 2014;134(6):1198–204. doi: 10.1016/j.thromres.2014.09.011 [published Online First: 2014/10/09] 25294589

[21] Eriksson BI, Dahl OE, Büller HR, et al. A new oral direct thrombin inhibitor, dabigatran etexilate, compared with enoxaparin for prevention of thromboembolic events following total hip or knee replacement: the BISTRO II randomized trial. *J Thromb Haemost* 2005;3(1):103–11. doi: 10.1111/j.1538-7836.2004.01100.x [published Online First: 2005/01/07] 15634273

[22] Eriksson BI, Dahl OE, Huo MH, et al. Oral dabigatran versus enoxaparin for thromboprophylaxis after primary total hip arthroplasty (RE-NOVATE II*). A randomised, double-blind, non-inferiority trial. *Thromb Haemost* 2011;105(4):721–9. doi: 10.1160/TH10-10-0679 [published Online First: 2011/01/13] 21225098

[23] Eriksson BI, Dahl OE, Rosencher N, et al. Oral dabigatran etexilate vs. subcutaneous enoxaparin for the prevention of venous thromboembolism after total knee replacement: the RE-MODEL randomized trial. *J Thromb Haemost* 2007;5(11):2178–85. doi: 10.1111/j.1538-7836.2007.02748.x [published Online First: 2007/09/04] 17764540

[24] Eriksson BI, Dahl OE, Rosencher N, et al. Dabigatran etexilate versus enoxaparin for prevention of venous thromboembolism after total hip replacement: a randomised, double-blind, non-inferiority trial. *Lancet* 2007;370(9591):949–56. doi: 10.1016/S0140-6736(07)61445-7 [published Online First: 2007/09/18] 17869635

[25] Ginsberg JS, Davidson BL, Comp PC, et al. Oral thrombin inhibitor dabigatran etexilate vs North American enoxaparin regimen for prevention of venous thromboembolism after knee arthroplasty surgery. *J Arthroplasty* 2009;24(1):1–9. doi: 10.1016/j.arth.2008.01.132 [published Online First: 2008/06/07] 18534438

[26] Agnelli G, Eriksson BI, Cohen AT, et al. Safety assessment of new antithrombotic agents: lessons from the EXTEND study on ximelagatran. *Thromb Res* 2009;123(3):488–97. doi: 10.1016/j.thromres.2008.02.017 [published Online First: 2008/05/20] 18485453

[27] Colwell CW Jr., Berkowitz SD, Davidson BL, et al. Comparison of ximelagatran, an oral direct thrombin inhibitor, with enoxaparin for the prevention of venous thromboembolism following total hip replacement. A randomized, double-blind study. *J Thromb Haemost* 2003;1(10):2119–30. doi: 10.1046/j.1538-7836.2003.00368.x [published Online First: 2003/10/03] 14521593

[28] Eriksson BI, Agnelli G, Cohen AT, et al. The direct thrombin inhibitor melagatran followed by oral ximelagatran compared with enoxaparin for the prevention of venous thromboembolism after total hip or knee replacement: the EXPRESS study. *J Thromb Haemost* 2003;1(12):2490–6. doi: 10.1111/j.1538-7836.2003.00494.x [published Online First: 2003/12/17] 14675083

[29] Bauer KA, Eriksson BI, Lassen MR, et al. Fondaparinux compared with enoxaparin for the prevention of venous thromboembolism after elective major knee surgery. *N Engl J Med* 2001;345(18):1305–10. doi: 10.1056/NEJMoa011099 [published Online First: 2002/01/17] 11794149

[30] Eriksson BI, Bauer KA, Lassen MR, et al. Fondaparinux compared with enoxaparin for the prevention of venous thromboembolism after hip-fracture surgery. *N Engl J Med* 2001;345(18):1298–304. doi: 10.1056/NEJMoa011100 [published Online First: 2002/01/17] 11794148

[31] Fuji T, Fujita S, Ochi T. Fondaparinux prevents venous thromboembolism after joint replacement surgery in Japanese patients. *Int Orthop* 2008;32(4):443–51. doi: 10.1007/s00264-007-0360-7 [published Online First: 2007/05/01] 17468868 PMC2532275

[32] Lassen MR, Bauer KA, Eriksson BI, et al. Postoperative fondaparinux versus preoperative enoxaparin for prevention of venous thromboembolism in elective hip-replacement surgery: a randomised double-blind comparison. *Lancet* 2002;359(9319):1715–20. doi: 10.1016/S0140-6736(02)08652-X [published Online First: 2002/06/07] 12049858

[33] Turpie AG, Bauer KA, Eriksson BI, et al. Postoperative fondaparinux versus postoperative enoxaparin for prevention of venous thromboembolism after elective hip-replacement surgery: a randomised double-blind trial. *Lancet* 2002;359(9319):1721–6. doi: 10.1016/S0140-6736(02)08648-8 [published Online First: 2002/06/07] 12049860

[34] Anderson DR, Dunbar MJ, Bohm ER, et al. Aspirin versus low-molecular-weight heparin for extended venous thromboembolism prophylaxis after total hip arthroplasty: a randomized trial. *Ann Intern Med* 2013;158(11):800–6. doi: 10.7326/0003-4819-158-11-201306040-00004 [published Online First: 2013/06/05] 23732713

[35] Hull RD, Pineo GF, Francis C, et al. Low-molecular-weight heparin prophylaxis using dalteparin in close proximity to surgery vs warfarin in hip arthroplasty patients: a double-blind, randomized comparison. The North American Fragmin Trial Investigators. *Arch Intern Med* 2000;160(14):2199–207. doi: 10.1001/archinte.160.14.2199 [published Online First: 2000/07/25] 10904464

[36] Hull RD, Pineo GF, Francis C, et al. Low-molecular-weight heparin prophylaxis using dalteparin extended out-of-hospital vs in-hospital warfarin/out-of-hospital placebo in hip arthroplasty patients: a double-blind, randomized comparison. North American Fragmin Trial Investigators. *Arch Intern Med* 2000;160(14):2208–15. doi: 10.1001/archinte.160.14.2208 [published Online First: 2000/07/25] 10904465

[37] Hur M, Park S-K, Koo C-H, et al. Comparative efficacy and safety of anticoagulants for prevention of venous thromboembolism after hip and knee arthroplasty. *Acta Orthopaedica* 2017;88(6):634–41. doi: 10.1080/17453674.2017.1361131 28787226 PMC5694808

[38] Venker BT, Ganti BR, Lin H, et al. Safety and Efficacy of New Anticoagulants for the Prevention of Venous Thromboembolism After Hip and Knee Arthroplasty: A Meta-Analysis. *J Arthroplasty* 2017;32(2):645–52. doi: 10.1016/j.arth.2016.09.033 [published Online First: 2016/11/09] 27823844 PMC5258767

[39] Lu G, Ades AE. Combination of direct and indirect evidence in mixed treatment comparisons. *Statistics in Medicine* 2004;23(20):3105–24. doi: 10.1002/sim.1875 15449338

[40] Lu GB, Ades AE. Assessing evidence inconsistency in mixed treatment comparisons. *Journal of the American Statistical Association* 2006;101(474):447–59. doi: 10.1198/016214505000001302

[41] Clark HD, Wells GA, Huet C, et al. Assessing the quality of randomized trials: Reliability of the Jadad scale. *Controlled Clinical Trials* 1999;20(5):448–52. doi: 10.1016/s0197-2456(99)00026-4 10503804

[42] Lumley T. Network meta-analysis for indirect treatment comparisons. *Statistics in Medicine* 2002;21(16):2313–24. doi: 10.1002/sim.1201 12210616

[43] Chung H, Lumley T. Graphical exploration of network meta-analysis data: the use of multidimensional scaling. *Clinical Trials* 2008;5(4):301–07. doi: 10.1177/1740774508093614 18697844

[44] Salanti G, Ades AE, Ioannidis JPA. Graphical methods and numerical summaries for presenting results from multiple-treatment meta-analysis: an overview and tutorial. *Journal of Clinical Epidemiology* 2011;64(2):163–71. doi: 10.1016/j.jclinepi.2010.03.016 20688472

[45] Turpie AGG, Schmidt A, Lassen MR, et al. RIVAROXABAN FOR THROMBOPROPHYLAXIS AFTER TOTAL HIP OR KNEE REPLACEMENT SURGERY: COMPARISON OF OUTCOMES OF THE XAMOS AND RECORD STUDIES. *American Journal of Hematology* 2014;89(6):E74–E75.

[46] Hosaka K, Saito S, Ishii T, et al. Safety of Fondaparinux Versus Enoxaparin After TKA in Japanese Patients. *Orthopedics* 2013;36(4):E428–E33. doi: 10.3928/01477447-20130327-17 23590781

[47] Bailar JC. The promise and problems of meta-analysis. *New England Journal of Medicine* 1997;337(8):559–61. doi: 10.1056/NEJM199708213370810 9262502

