# Supplementary figures and images for: Efficacy and safety of anticoagulants for postoperative thrombophylaxis in total hip and knee arthroplasty: A PRISMA-compliant Bayesian network meta-analysis
Source: PLoS One. 2021 Jun 17;16(6):e0250096. doi: 10.1371/journal.pone.0250096 (PMC8211213; doi:10.1371/journal.pone.0250096)

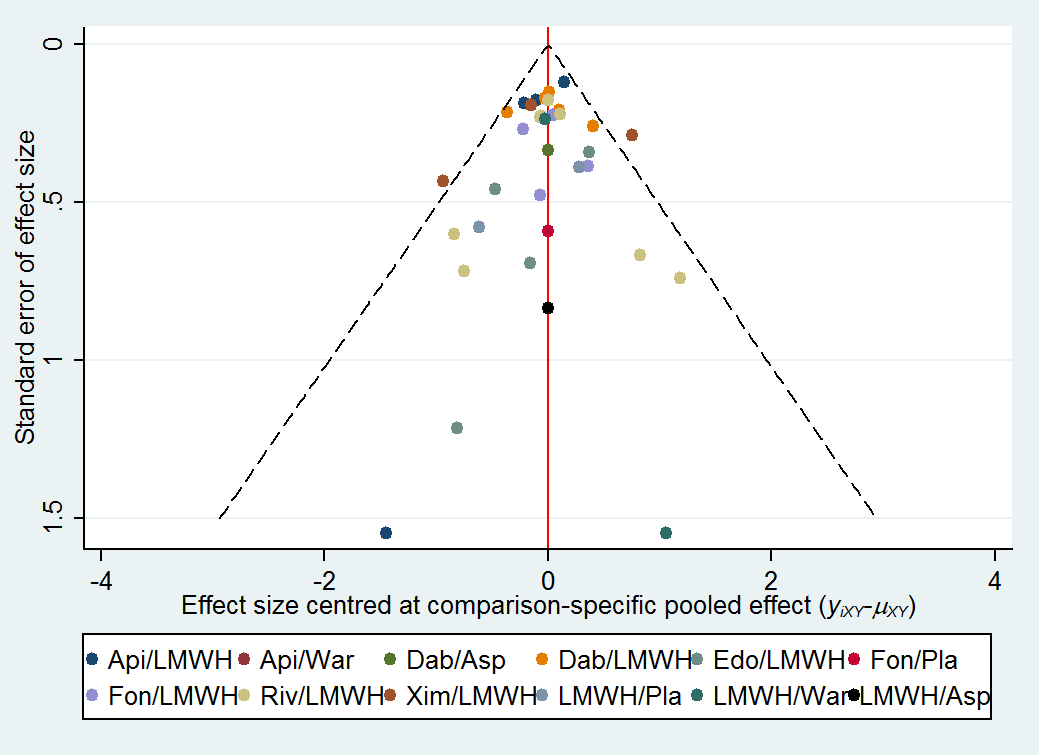

Supplement: S1 Fig — (TIF) [file pone.0250096.s001.tif]

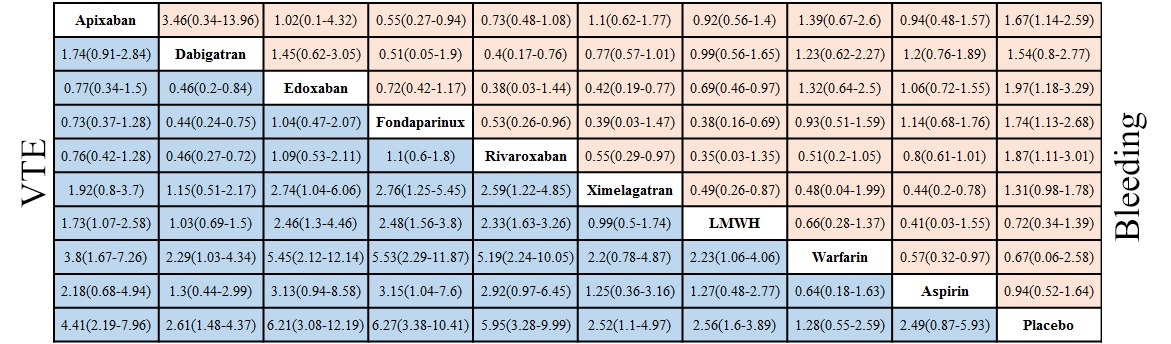

Supplement: S2 Fig — No significant difference was observed between the direct and indirect comparisons. (TIF) [file pone.0250096.s002.tif]

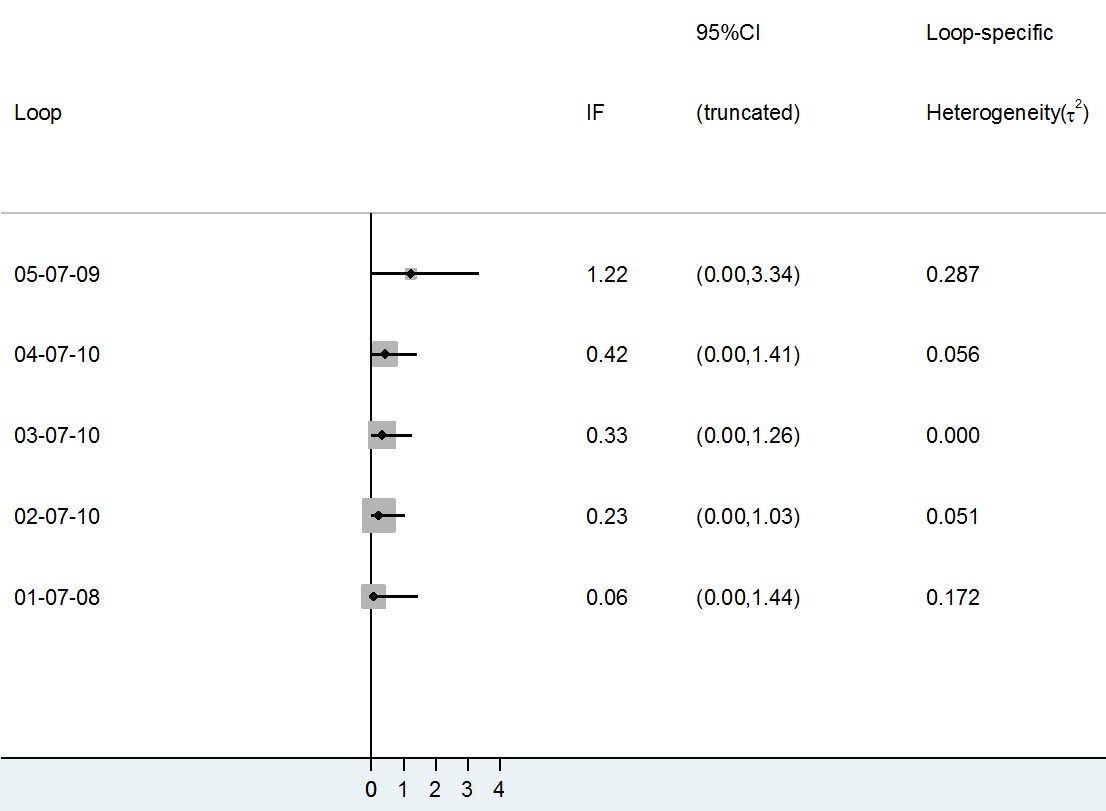

Supplement: S3 Fig — Publication bias was not significant across the selected citations. (TIF) [file pone.0250096.s003.tif]

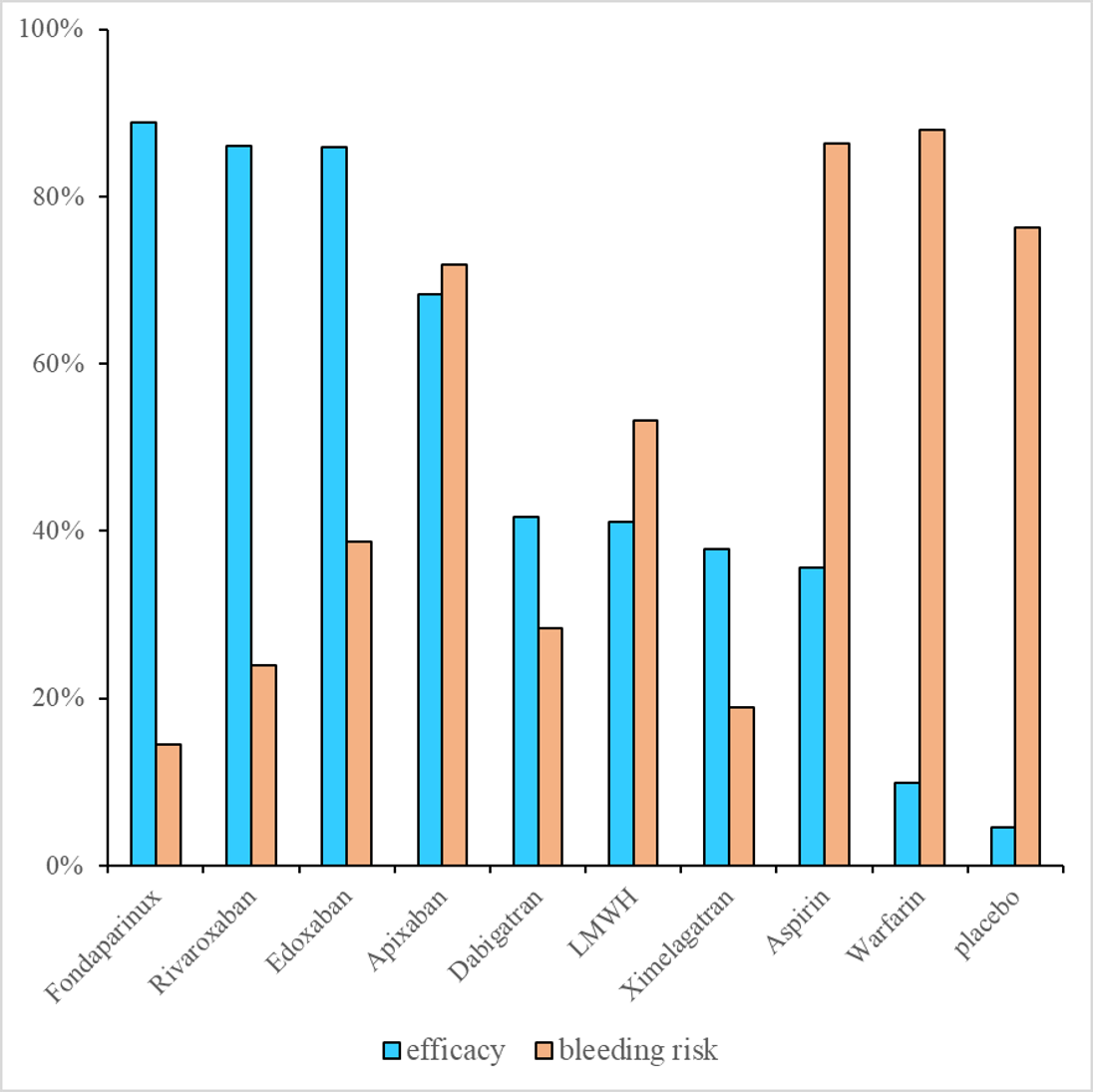

Supplement: S4 Fig — (TIF) [file pone.0250096.s004.tif]

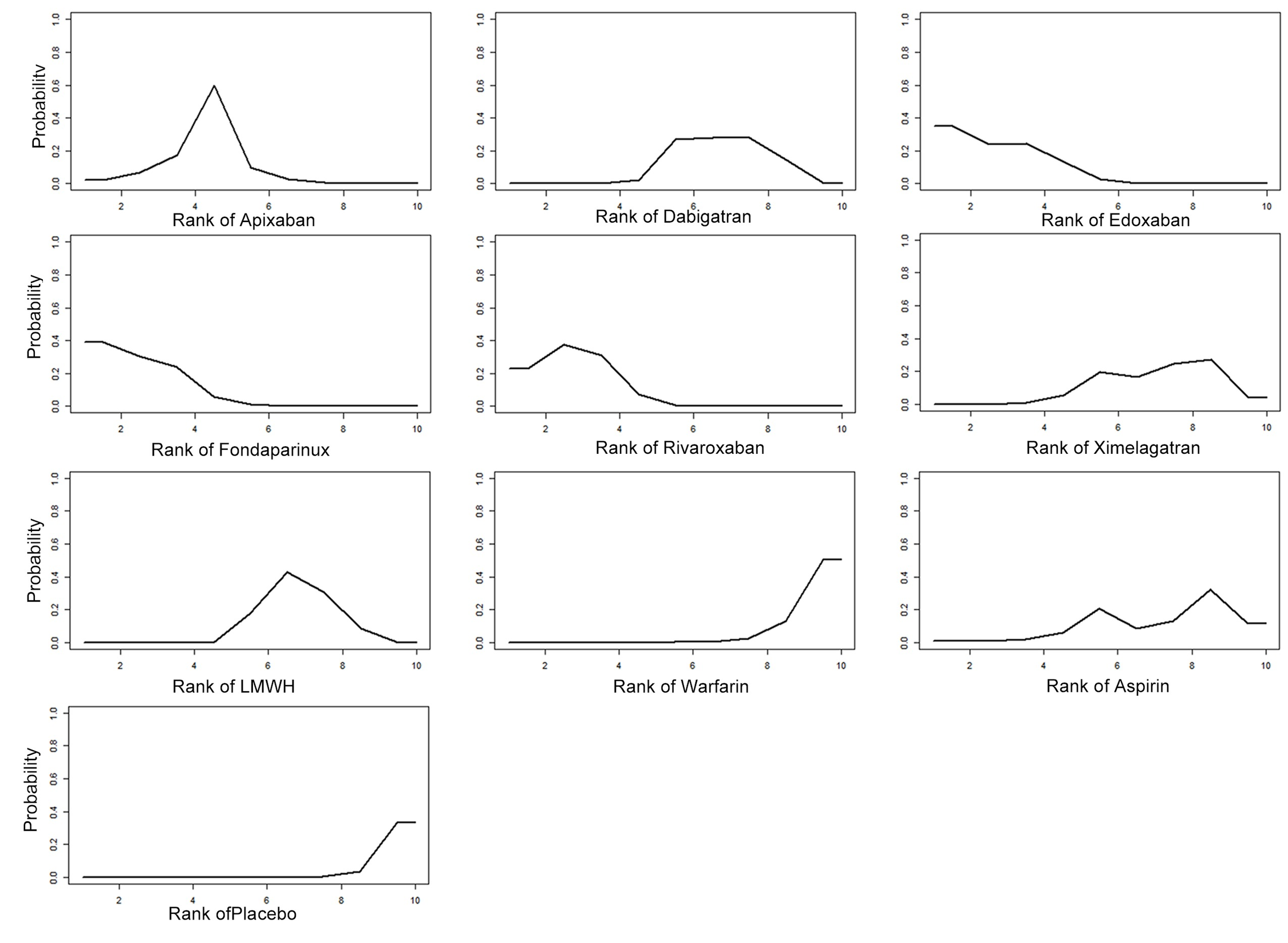

Supplement: S5 Fig — Treatments with a higher SUCRA position for VTE prophylaxis were associated with larger probabilities of better outcomes. (TIF) [file pone.0250096.s005.tif]

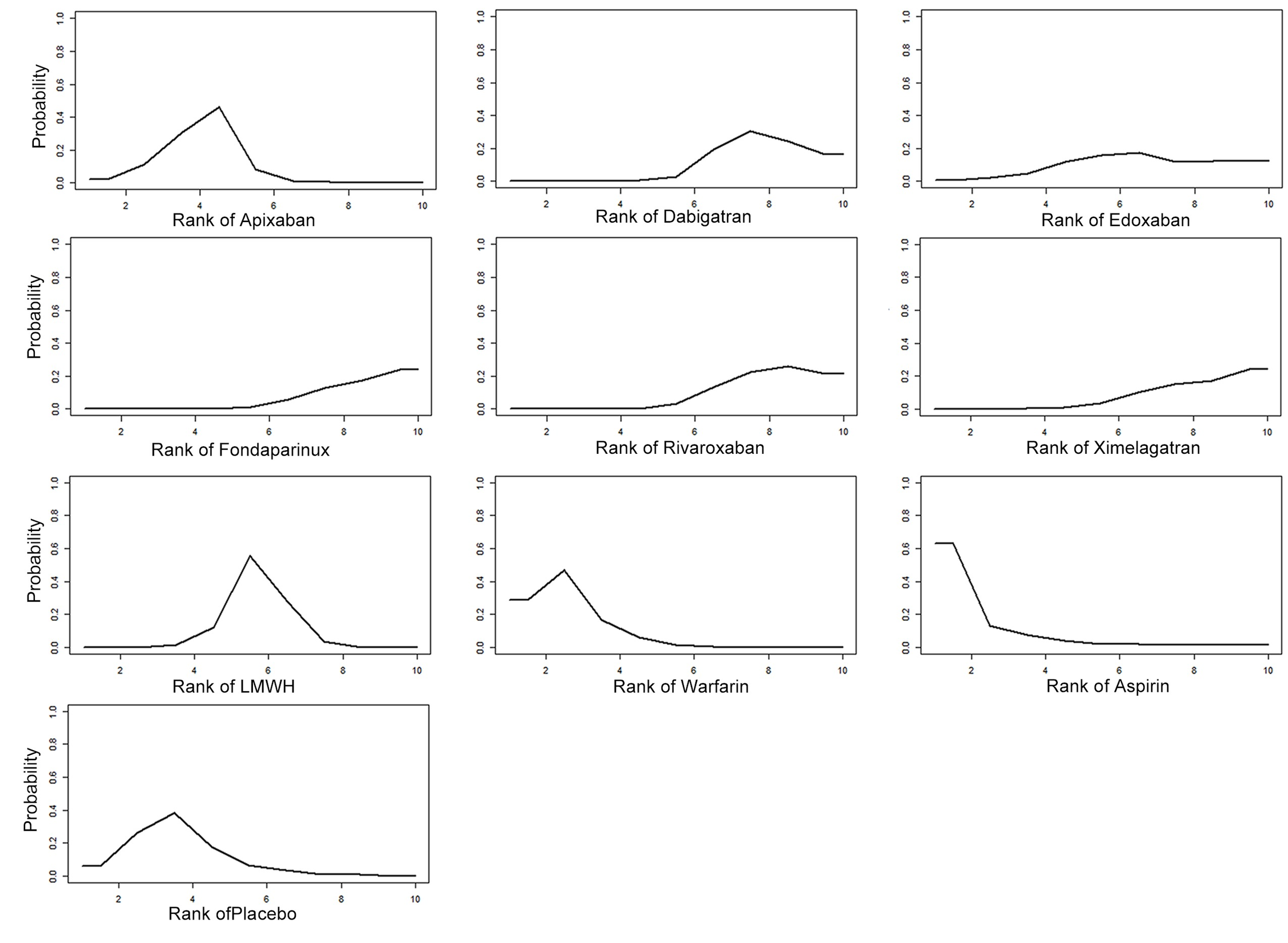

Supplement: S6 Fig — A lower SUCRA position for side effects (major or clinically relevant bleeding) indicated a higher priority of safety. (TIF) [file pone.0250096.s006.tif]
