# Supplementary material for: Efficacy and safety of anticoagulants for postoperative thrombophylaxis in total hip and knee arthroplasty: A PRISMA-compliant Bayesian network meta-analysis
Source: PLoS One. 2021 Jun 17;16(6):e0250096. doi: 10.1371/journal.pone.0250096 (PMC8211213; doi:10.1371/journal.pone.0250096)
Supplement: S1 File — (DOC) [file pone.0250096.s007.doc]

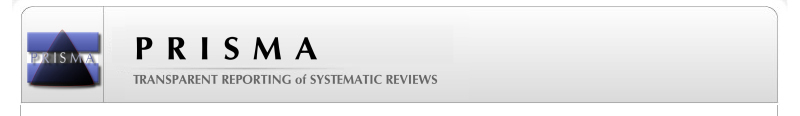
**PRISMA 2009 Flow Diagram**

**Screening**

**Included**

**Eligibility**

**Identification**

Records identified through database searching
(n = 8062)

Records after duplicates removed
(n =4591)

Records screened
(n =49)

Records excluded
no relevance (n =4542)

Full-text articles assessed for eligibility
(n =49)

Full-text articles excluded (n =14) for:

study type (n =9),

non-interest(n =5)

Studies included in quantitative synthesis (meta-analysis)
(n =35)
